# OM-101 Decreases the Fibrotic Response Associated with Proliferative Vitreoretinopathy

**DOI:** 10.1155/2017/1606854

**Published:** 2017-10-04

**Authors:** Zeev Dvashi, Keren Ben-Yaakov, Tamir Weinberg, Yoel Greenwald, Ayala Pollack

**Affiliations:** Kaplan Medical Center-Rehovot, Affiliated to Hadassah-Hebrew University of Jerusalem, Rehovot, Israel

## Abstract

**Purpose:**

This study aimed to investigate the effect of OM-101 on the fibrotic response occurring in proliferative vitreoretinopathy (PVR) in an animal model.

**Methods:**

Antifibrotic effect of OM-101 was investigated *in vivo*. As control, eight weeks old c57black mice underwent intravitreal injection with Hepes (group A) or dispase (0.3 units), to induce retinal detachment (RD) and PVR. The dispase-injected mice were randomly divided into two groups B and C (*N* = 25 mice); in group C, the eyes were treated with intravitreal injection of OM-101 (3 *μ*l), and group B with PBS, as a control. After additional five days, mice were injected with the same initial treatment. Three days later, mice were euthanized, and the eyes were enucleated and processed for histological analysis.

**Results:**

Intravitreal injection of dispase caused RD in 64% of the mice in group B, and 93% of those mice had PVR. Only 32% of mice treated with OM-101 and dispase (group C) developed RD, and only 25% of those developed PVR.

**Conclusions:**

OM-101 was found effective in reducing the incidence of RD and PVR maintaining the normal architecture of the retina. This study suggests that OM-101 is a potentially effective and safe drug for the treatment of PVR patients.

## 1. Introduction

Proliferative vitreoretinopathy (PVR) is a severe complication of rhegmatogenous retinal detachment (RD) [1, 2]. PVR occurs in 8–10% of the eyes undergoing repair of RD and represents 10–45% of open global trauma in USA [3, 4]. In spite of repeated surgeries, it may result in visual loss. Current therapy modalities that aimed to limit or reverse PVR have failed to achieve significant clinical benefits.

PVR is a fibrotic process characterized by proliferation and migration of cells, inducing formation of contractile epi-retinal membranes [5]. The primary cells involved in epi-retinal proliferation are retinal pigment epithelial (RPE) cells. Epithelial to mesenchymal transition (EMT) of RPE cells to fibroblasts is the cellular event that underlies PVR. A key protein in the EMT process is transforming growth factor beta-activated kinase 1 (TAK1) that acts in the noncanonical pathway of TGF-*β* [6, 7]. Previous studies performed in our laboratory demonstrated that TAK1 acts as a critical player in the regulation of RPE cells during EMT. By applying TGF-*β*1 on human ARPE-19 cells in culture and utilizing various experimental approaches, we show that inhibition of TAK1 by a specific inhibitor, 5Z-7 oxozeaenol, reduces cell migration, alpha-smooth muscle actin (*α*-SMA) expression, and cell motility, all of which are considered hallmarks of fibrosis during PVR [8]. TAK1 inhibitor 5Z-7 oxozeaenol contains a cis enone at its 6′ to 8′ position that could function as Michael acceptor for an appropriately positioned thiol functional group of a cysteine residue of TAK1 to form an irreversible kinase-inhibitor complex [9]. Lastly, utilizing collagen contraction assay and TAK1 inhibitor, we demonstrate that TAK1 is a general regulator of the fibrotic response in RPE cells.

Those results point to the prominent roles of TAK1 in inflammation and fibrosis events, such as PVR [8].

This study aimed at examining the use of OM-101 (formulation of 5Z-7 oxozeaenol) in an animal model, as a new horizon in the treatment of PVR.

## 2. Material and Methods

### 2.1. Proliferative Vitreoretinopathy (PVR) Induction and Treatment

PVR was induced by intravitreal injection of dispase as previously described [10]. Briefly, mice were anesthetized by intraperitoneal injection of ketamine (100 mg/kg) and xylasin (10 mg/kg) mixture. Then, 3 *μ*l of Dispase II (10 mg/ml, Sigma number D493, dissolved in Hepes/KOH pH = 7.4, 50 mM NaCl buffer) was injected intravitreally to the right eye. As control, mice in group A were injected with 3 *μ*l of the Hepes solution (*N* = 5 mice). Three days later, dispase-injected mice were randomly divided into two additional groups (groups B and C, *N* = 5 mice each group). Group C was treated with an intravitreal injection of OM-101 (3 *μ*l, 1 *μ*M), while groups A and B were injected with PBS; all injections were to the right eye only. After additional five days, mice were injected with the same treatments. Three days later, the mice were euthanized and the eyes were enucleated and processed for histological analysis. Blood was collected to evaluate the safety of OM-101 tissue samples including the brain, liver, lung, spleen, kidney, and the left eye that were histology evaluated for abnormal morphology.

All experiments were compliant with the ARVO Statement for the Use of Animals in Ophthalmic and Vision Research and approved by the Institutional Animal Care and Use Committee at Hebrew University of Jerusalem.

### 2.2. OM-101 Preparation

1 mg of 5Z-7 oxozeaenol was dissolved in 276 *μ*l of DMSO for a final concentration of 10 mM or 3.623 *μ*g/*μ*l and further diluted 1 : 2000 in PBS for a final concentration of 5 *μ*M or to 1.811 ng/*μ*l. 5 *μ*l (9 ng) of this dilution was used for injections.

### 2.3. Funduscopy and Optical Coherence Tomography (OCT)

Mice were anesthetized by intraperitoneal injection of ketamine (100 mg/kg) and xylasin (10 mg/kg) mixture. The eyes were topically anesthetized with one drop of Localin drops. The corneas were kept moist with regular application of 2.5% methylcellulose, and pupils were dilated with Tropicamide 1%. Eyes were examined using the Micron III retinal imaging system (Phoenix Research Labs, CA, USA), and raw images were adjusted for levels, enhanced contrast, and sharpened by applying an unsharp mask (100%, 2px, 0) using Photoshop CS6 (Adobe, Ca, USA).

### 2.4. Histological Staining

Nine *μ*m paraffin embedded sections of the eye tissues were stained with hematoxylin and eosin (H&E) as described in previous reports [11]. Histological evaluation was performed to assess the RD and PVR. The severity of PVR was defined by infiltration of white blood cells such as macrophages or B cells (0—absence of cells, 1—low frequency, 2—moderate frequency, and 3—high frequency), edema, and the appearance of pigment cells in the retina (cell migration).

### 2.5. Blood Marker Assessment

Blood was collected into K3 EDTA tubes and underwent examination and analysis according to standard procedures. Liver functionality was manifested by alanine transaminase (ALT) and aspartate aminotransferase (AST) markers. Pancreas functionality was manifested by glucose levels and kidney by NA and urea levels.

### 2.6. Statistics

Statistics were computed using Student's *t*-test and two-tailed distribution. Values of *P* < 0.05 were considered significant.

## 3. Results

**Figure 1:**
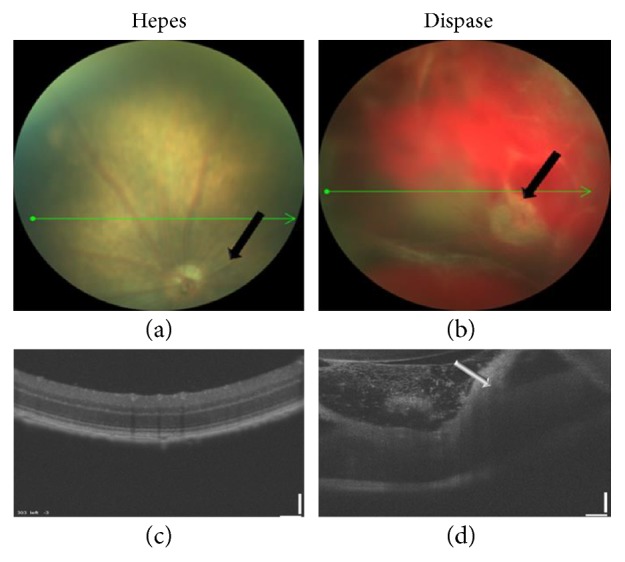
Funduscopy and OCT in mice eyes after dispase injection. Mice injected with Hepes only (control) demonstrated normal retina (a). Mice injected with dispase demonstrating RD with fibrotic tissue in front of the retina (b). Normal retina on OCT (c) and detached retina with loss of normal appearance of the retina (d).

**Figure 2:**
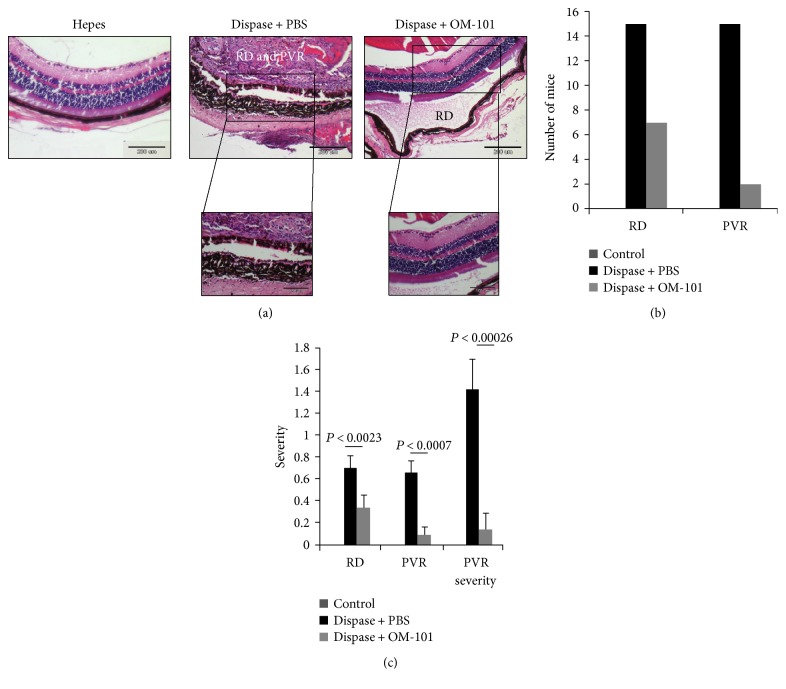
Histological staining (H&E) of induced RD and PVR in retina treated with OM-101. (a) Histological staining (H&E) demonstrated the following: left (control/Hepes), normal architecture; middle (dispase + PBS), abnormal morphology with inflammatory cells, fibroblasts, and pigment stain RPE cells through the retina denoting PVR; and right (dispase + OM 101), OM 101 treatment maintained the normal structure of the retina. Scale bar 100 *μ*m or 200 *μ*m. (b) Number of mice developing RD or PVR. (c) Severity score of the mice retina measured by thickness of the RD, number of inflammatory cells, loss of normal structure, and the appearance of RPE cells inside the retina (*N* = 25). Statistics were computed using Student's *t*-test (two-tailed distribution equal variance). Data is expressed as the mean ± SD.

In this paper, we have used dispase to generate retinal detachment and subsequently proliferative vitreoretinopathy (PVR). Dispase, a proteolytic enzyme able to harvest and culture cells due to its ability to cleave the basal membrane in various tissues, can be used as an intravitreal injection material to induce PVR in the eyes of mice. Mice were injected with dispase as described by Tan et al. [12] and underwent fundus and OCT analyses to verify RD formation (Figure 1). Mice injected with Hepes (group A) demonstrated a normal retina, whereas dispase-treated mice showed RD seen in both fundus (Figure 2(a), upper) and OCT analyses (Figure 2(a), lower). Dispase-injected mice were randomly divided into two groups: PBS-injected (group B) and OM-101-injected (group C) mice. PVR-like traces could be clearly found after 10 days in group B. H&E-stained sections showed marked proliferative membranes, retinal detachment, serous fluid between the RPE and the sensory retinas, and destructed retinas (Figure 2(a), middle). Furthermore, infiltration of the RPE cells into the center of the retina was detected in group B, thus demonstrating increased migratory capacity of these cells. In contrast, mice injected with OM-101 demonstrated only RD, with normal retinal structure and clear form RPE cells (Figure 2(a), right). Quantitative evaluation of mice with RD or PVR from the different groups demonstrated that intravitreal injection of dispase triggers RD in 64% of mice in group B, and 93% of those mice had PVR. In contrast, only 32% of OM-101-treated mice (group C) developed RD, and 25% of those developed PVR (Figure 2(b)). In mice injected only with Hepes and PBS (group A), neither RD nor PVR was found. Importantly, the severity of the PVR measured in the mice of group B was significantly higher than in mice treated with OM-101 in all parameters (Figure 2(c)), demonstrating marked infiltration of macrophages and a fibrotic response.

**Figure 3:**
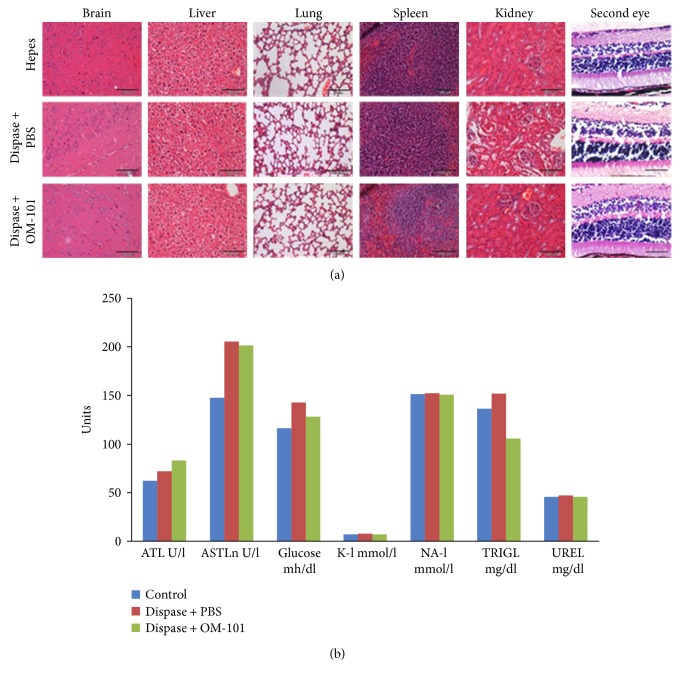
Safety studies of OM-101 organs (a) and blood (b) were collected from all groups. All blood parameters were found normal compared to the control group. No abnormalities were found in the histological section. Scale bar 100 *μ*m.

These results demonstrate the positive effect of OM-101 in reducing the occurrence of PVR in the dispase model system. To consider OM-101 as an optional therapeutic avenue for PVR, safety experiments were performed. Injection of mice intravitreally with OM-101 did not affect the morphology of different organs such as the brain, liver, lung, spleen, and kidney (Figure 3(a)). Correspondingly, blood markers from OM-101-treated mice demonstrated normal liver, kidney, and pancreas functions following OM-101 injection.

## 4. Discussion

This study shows that treatment with OM-101 can inhibit and prevent PVR by preserving normal architecture in dispase-induced RD, manifested by decreased number of proliferating RPE cells and by reduced fibrotic response.

During retinal detachment, the RPE cells disseminate into the vitreous cavity and onto the retinal surface, as well as proliferate and migrate throughout the retina and towards the vitreous, transdifferentiate into mesenchymal-like *α*-smooth muscle actin- (*α*-SMA-) positive cells that produce extracellular matrix, and contribute to the accumulation of fibrous scar tissue. Such transdifferentiation is considered to be a process of epithelial-mesenchymal transition (EMT), a program of differentiation whereby cells lose their epithelial morphologic features and acquire more mesenchymal-like morphologic features in association with expression of mesenchymal markers such as *α*-SMA [13].

Although various growth factors are reportedly involved in the pathogenesis of PVR, transforming growth factor *β* (TGF-*β*) is the key regulator for those processes [1, 3, 14]. A key protein in the process is TAK1 that acts in the noncanonical pathway of TGF-*β* [6, 7].

Even though TAK1 activation was first identified as a mediator of TGF-*β*1 signaling, it is well-known today that TAK1 can also be activated by various other stimuli, including environmental stress and proinflammatory factors such as tumor necrosis factor- (TNF-) *α*, interleukin- (IL-) 1, and lipopolysaccharides (LPS) [15]. Previous work performed in our laboratory has demonstrated that stimulation of RPE cells with TGF-*β*1 increases *α*-SMA expression, cell migration, and cell contractility, all of which are EMT features. Remarkably, addition of TAK1 inhibitor abolished all these processes, suggesting that the outcome of the TGF-*β*-induced response in RPE cells is TAK1-dependent. Those results point to the prominent roles of TAK1 in inflammation and fibrosis events, such as PVR inhibiting [8].

The use of a specific inhibitor for TAK1 abolished all EMT characteristics that were induced in RPE cells by TGF-*β* [8]. Furthermore, we demonstrated that TAK1 inhibitor reduced the activation of both the canonical and noncanonical pathways of TGF-*β*1 signaling [8]. Those *in vitro* results stood as the basis of this current research examining the effects of OM-101 on PVR induced in mice.

In PVR, when RPE cells become dislodged into the vitreous cavity or beneath the neurosensory retina, they experience an environmental change regarding exposure to cytokines and growth factors, and their normal cell-cell and cell-matrix interactions are disrupted. This process causes enhanced cell migration, high levels of *α*-SMA expression, and increased contractility [16]. Our results demonstrate that by inhibiting TAK1 activity, these processes are significantly attenuated *in vitro*. Besides, specific inhibition of TAK1 maintains the quiescent and naive form of the RPE cells [17]. Furthermore, we have previously shown that the role of TAK1 in the process of EMT is not restricted to TGF-*β*1 signaling, rather it is a general function, as established by the collagen contraction assay. The event of impaired RPE contractility in the presence of TAK1 inhibitor occurs in full serum medium where all chemokines and cytokines play a part, thus demonstrating a TAK1 general role in the process of EMT in RPE cells.

Several studies investigated the roles of TGF-*β* signaling in PVR; however, these papers did not show significant reduction in the complication underlying PVR following perturbations of TGF-*β* signaling [18, 19]. Furthermore, TGF-*β* is a pivotal player in numerous molecular events; thus, inhibition of this growth factor might result in severe pathologies. In contrast, OM-101 that affects downstream proteins in the TGF-*β* cascade is safer and inhibits specific events related to fibrosis.

In this study, we used the dispase model system to investigate OM-101 effects during PVR [10]. Dispase initiated the development of PVR without the addition of exogenous cells, growth factors, or cytokines typically found in PVR membranes. A cascade of events was triggered by dispase, causing native cells and factors to produce PVR [12]. The dispase model of PVR is technically easy to perform, permitted a clear view of the retina, and had a high success rate in development of PVR.

There is an emerging complex picture around the biochemical and the molecular events that drive the pathogenesis of PVR. It is becoming clearer that interplay exists between various cytokines and growth factors, matrix proteins, and the different cell types that drive the undesirable formation of epi-retinal membranes [20]. As we and others showed, a key growth factor involved in this process is TGF-*β*. Thus, proper inhibition of this pathway may be effective to reduce the complications of PVR. This fundamental understanding, added to the unsatisfactory success rate of surgery, is aiding in identifying the efficacy of different agents that can block the cellular events intrinsic to PVR. Still, we cannot rule out that OM-101 in consequence modifies the early inflammatory response but its impact on PVR has a need to be further elaborated.

Our data is demonstrating for the first time the positive effect of OM-101 reducing the occurrence of PVR in an animal model. PVR develops as a complication of RD or open globe trauma (OGT). PVR occurs in about 10% of patients undergoing primary RD surgery and leads to RD surgical failure. In OGT, an average of 25% of patients may develop PVR. The results of surgery are often unfavorable accompanied by poor visual outcome. Thus, it should be noted that even though OM-101 effect on PVR was tested in this paper during the progression of the disease, OM-101 treatment can be considered as a potential prophylaxis treatment after RD or OGT. This may bring a new horizon in the treatment of PVR, and OM-101 may be used as a novel therapeutic approach for the treatment of PVR, thus preventing subsequent blindness.
